# Pyrrolizidine alkaloids quantified in soil and water using UPLC-MS/MS†

**DOI:** 10.1039/c9ra05301h

**Published:** 2019-09-25

**Authors:** Jawameer R. Hama, Bjarne W. Strobel

**Affiliations:** Department of Plant and Environmental Sciences, University of Copenhagen Thorvaldsensvej 40 1871 Frederiksberg Denmark jawameer@plen.ku.dk

## Abstract

Pyrrolizidine alkaloids (PAs) are produced in plants as defence compounds against insects. PAs present a serious health risk to humans and livestock; therefore it is necessary to have a validated analytical method to monitor PAs in the environment. The objective of this work is to present an UPLC-MS/MS method for quantification of PAs in environmental samples of both soil and water. A fast, reliable, and sensitive approach is developed to identify and quantify PAs in soil and water. Sample preparation was performed by clean-up and pre-concentration of the samples using MCX solid phase extraction cartridges with full optimization, and then PAs were determined by UPLC coupled with TQ-MS. In the liquid chromatography, most of the parameters were optimized and tested including gradient time, solvents, additives, and pH of the mobile phases and flow rate. In addition, the MS parameters of cone voltage, desolvation temperature, cone flows, and collision energy were optimized. The instrument limit of detection (2–7 μg L^−1^) and limit of quantification (5–9 μg L^−1^) were determined experimentally, and the method was linearity validated up to 1000 μg L^−1^. The method was applied to analyse soil and surface water samples collected in April and May 2018 in Vejle, Borup, and Holte, Denmark. In total, 15 PAs were quantified and reported for the first time in environmental samples, in a range of 3–1349 μg kg^−1^ in soil and 4–270 μg L^−1^ in surface water.

## Abstract 2

Pyrrolizidine alkaloids quantified in soil and water.

## Graphical abstract

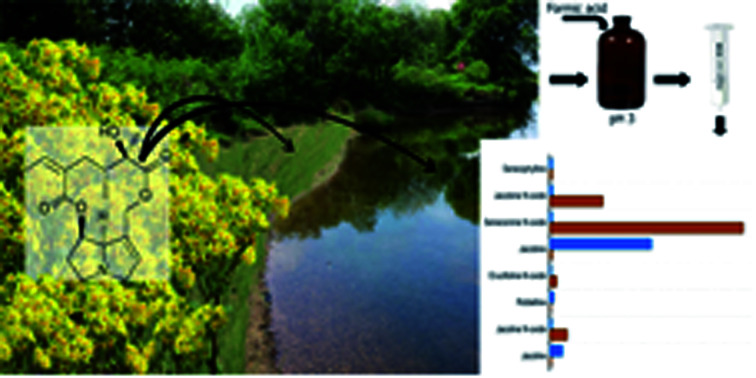

## 1. Introduction

Pyrrolizidine alkaloids (PAs) are secondary plant metabolites, consisting of nitrogen between two fused five-membered rings. PAs are esters of hydroxylated methyl pyrrolizidines, with a necine base and necic acid moiety that can be either saturated or unsaturated with a double bond between the 1 and 2 positions.^1^ PAs are classified into three classes: retronecine, otonecine and platynecine. They exist as either the free base (PA, tertiary amine) or N-oxide (PANO) form.^2,3^ So far, several hundred PAs have been found in nature and their structures determined.^4^ Retronecine and otonecine types have a 2-double bond in the necine bases, which are hepatotoxic and may lead to cancer in humans. However the platynecine type has a saturated necine base.^5–7^ Plants use PAs mostly as a defence mechanism against insects. It is estimated that approximately 3% of all flowering plants contain at least one PA. PAs are found in many plant species in the Asteraceae, Boraginaceae and Fabaceae families.^8,9^ PAs are among the most abundant group of natural toxins produced by plants.^10,11^

Toxicity of individual PAs is related to the chemical structure and physical properties.^7^ Cases of human exposure to PAs originate from direct consumption of PA-containing teas, herbal dietary and supplements.^9^ Moreover, exposure of PAs is likely by consuming tea, honey and animal product s which are contaminated with PAs.^7,12^ Due to safety hazards, some guidelines and regulations were issued to limit daily intake. These regulation are for particular cases and cannot be used for all situations.^9,12–14^ German Federal Institute for Risk Assessment (BfR) recommended 0.007 μg PAs intake for humans per kg bodyweight.^12^ While EFSA chose 237 μg per kg bodyweight as a new reference point for MDL10 calculation from riddelliine's toxicity study, and corresponds to a maximum intake of 0.024 μg PA/PANO per kg bodyweight.^9^

**Fig. 1:**
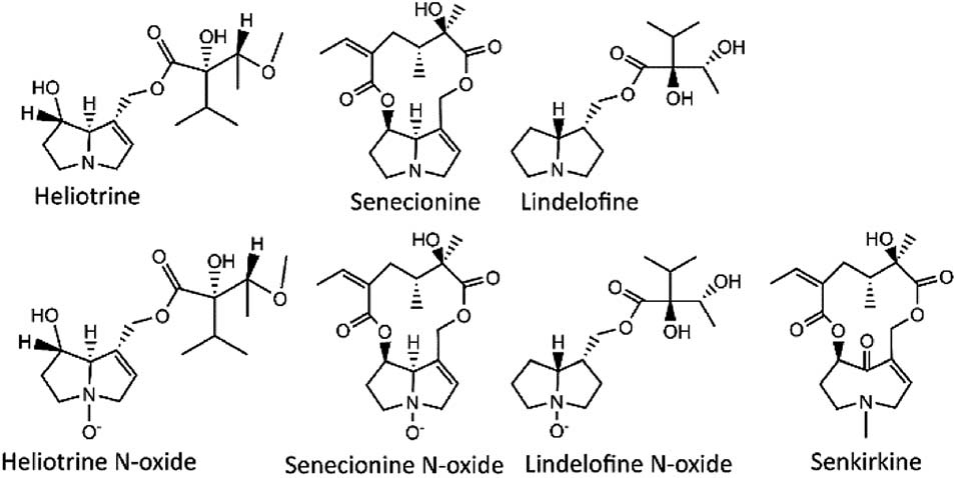
Structures of selected types of pyrrolizidine alkaloids.

Previous analytical methods are optimized to detect PAs in a certain commodities. In particular, LC-MS methods optimized to detect PAs in foods, is more for quality control of food and of foodstuffs.^1,10,15–17^ The UPLC coupled with MS has been used to detect a trace-level of organic pollutants with high selectivity and sensitivity, with shorter run time and sharper peaks, this may reduce or eliminate the matrix effects issue.^18–20^ High resolution MS can provide accurate mass information that resulted in enhanced application of the instrument.^1,21^ Quantification and target analysis can be processed using high MS resolution with multiple reaction monitoring (MRM) transitions, however the exact masses is required.^22^ MRM transition is good for quantification of PAs, as they have more common fragmentation patterns and produce similar product ions.^17,21,23^ Cases of PA poisoning cannot be eliminated due to the lack of accurate analytical methods for a wider range of sample types. Thus, a robust analytical method to determine PAs (Fig. 1) in complex matrices is still missing.^24^

Here, we report a quick and robust UPLC-MS/MS method to monitor and quantify PAs in a wide range of environmental samples such as plant, soil and water. The method enables analysis of small and large volumes of environmental samples after fast SPE to pre-concentration and clean-up the samples.

## 2. Experimental

### 2.1 Chemicals and reagents

Methanol (MeOH) (MS grade), acetonitrile (MeCN) (MS grade), formic acid (FA) (MS grade), ammonium formate (MS grade), acetone (HPLC grade), sulfuric acid, hydrochloric acid (Analytical reagent grade) and caffeine (internal standard, for spiking experiments and method validation) were purchased from Sigma-Aldrich (Steinheim, Germany). Glass fibre (SiO_2_, particle size 0.2–0.8 mm) was purchased from Merck (Darmstadt, Germany). Certified reference materials of heliotrine, monocrotaline, monocrotaline N-oxide, senecionine, senecionine N-oxide, erucifoline, erucifoline-N-oxide, retrorsine, jacobine, jacobine N-oxide senkirkine, heliotrine N-oxide, echimidine, echimidine N-oxide, purchased from Phytolab (Vestenbergsgreuth, Germany), also lindelofine, lindelofine N-oxide purchased from AKos Consulting & Solutions (Steinen, Germany). Oasis MCX 6 cc, 150 mg Sorbent, 30 μm particle size purchased from Waters (Milford, USA). MilliQ water (resistivity 18.2 Mohm × cm, TOC less than 1 μg L^−1^) was produced in-house with a type I ultrapure water purification system from ELGA-Veolia LabWater (High Wycombe, UK).

### 2.2 Soil and water samples

Two surface water, three topsoil and one subsoil samples were collected in amber glass bottles in April and May 2018 in Vejle, Borup and Holte, Denmark (coordinates: N 55°41′00.0, E 9°25′00.0: N 55°27′00.0, E 12°00′00.0; N 55°49′00.0, E 12°30′00.0, respectively). The areas had Ragwort (*Senecio jacobaea* L.) plants in densities 20–28 plants per square meter as a source of PAs. Water samples were filtered with filter paper (Whatman® quantitative-Grade 42) to remove any suspended particles and acidified to pH 3 with diluted FA. Soils were sieved on 0.2 mm to remove the coarse sand and gravel, and visible plant roots removed. All samples were stored at −20 °C prior to extraction, in most cases the extraction was performed within 48 h after sampling. Each time, 10 g soil or 500 mL water were collected in duplicate, and stored at −20 °C prior to the analysis.

### 2.3 Sample preparation and extraction

#### 2.3.1 Soil extraction

Accurately 2.50 g of soil weighted into 25 mL centrifuge tube and added 10 mL MeOH, sonicated for 15 minutes and centrifuged for 10 min at 8000 rpm (2100*g*). The supernatant was collected for analysis. This extraction was repeated. After that, 10 mL of MeOH : acetone (85 : 15 v/v%) solvent used for a third and fourth extraction. The extraction efficiency was approved with 1 mL of the fourth aliquot of MeOH : acetone (85 : 15 v/v%) extract tested directly by UPLC-MS/MS method, and no PAs were detected. Finally, the three extracts were combined, centrifuged and filtered with a 0.45 μm PTFE Membrane filter, and then passed through MCX SPE.

#### 2.3.2 SPE procedure

MCX SPE cartridges were optimized for efficiency, including pH adjustment of the sample, acid wash solution and acid concentration, eluent volume and sample volume. The SPE cartridge was conditioned with 5 mL MeOH followed by 5 mL H_2_O. Then acidified (pH 3) soil extracts (30 mL) and water samples (500 mL) were passed through the cartridge with a 10 mL min^−1^ flow rate, and washed with 5 mL 0.065 mM formic acid. The PAs were eluted with 5 mL 50% MeOH and 10 mL methanol–10% ammonia (3 : 1, v/v). The eluates were combined and dried under gentle nitrogen flow in a heating block at 40 °C. The dried extract was dissolved in 1 mL 40% MeCN and filtered through 0.2 μm PTFE membrane filter prior to analysis.

### 2.4 Instrumentation and analytical conditions

#### 2.4.1 Chromatography

A Waters Acquity UPLC I-Class module was used for chromatographic separation, equipped with a 2.1 mm × 100 mm Acquity UPLC BEH C18 Column, particle size 1.7 μm (Waters, Milford). To improve the sensitivity of the method, the effect of gradient ramp, flow rate, mobile phase composition (MeCN and MeOH), pH (acid pH = 2.7 and base pH = 8) and additives (FA and NH_4_OH with NH_4_HCO_2_) were tested. Three gradients were tested for eluent ramp for both MeCN and MeOH. Gradient 1: 0–0.5 min 1% B, 5.5 min 99% B, 8 min 99% B, 8.1 min 1% B, 12 min 1% B, the total run time is 12 minutes. Gradient 2: 0–0.5 min 1% B, 7.5 min 99% B, 10 min 99% B, 10.1 min 1% B, 14 min 1% B, the total run time is 14 minutes. Gradient 3: 0–0.5 min 1% B, 9.5 min 99% B, 14 min 99% B, 14.1 min 1% B, 18 min 1% B, the total run time was 18 minutes. Five different flow rates (0.25–0.6 mL min^−1^) were tested to optimize the column pressure, and minimise the run time, without co-eluting the PAs. Different buffer system of 0.001 and 0.01% FA and 1, 3, and 10 mM ammonium formate with pH adjusted to 5 were used. The robustness of the methods was tested with different LC mobile phase compositions. To protect the MS from early eluting impurities, remaining salts and eluates during the cleaning step, the method event was set to direct the LC flow to waste outside ion trace windows. A mixture of 100 μg L^−1^ PAs standards was used to evaluate peak capacity, peak shape, ions traces, S/N ratio and relative standard deviation, in duplicate injections. Peak capacity describes the number of peaks that can be separated during a gradient. To calculate peak capacity the following equation is used: *P* = 1 + (*t*_G_/*w*), where *t*_G_ is the gradient time, *w* is peak width for a gradient separation expressed in time (min).^25^ Peak width represent retention dimensions (time) parallel to the baseline, it is measured at the base of the peaks by adjusting the peak base intercepted by the tangents drawn to both sides of the peak.^26^

In the optimized method, the column temperature was set to 35 °C. LC mobile phase was composed of A (water + 0.01% FA) and B (acetonitrile + 0.01% FA). Gradient conditions were: 0–4 min 10% B, 7 min 20% B, 10 min 50% B, 15 min 90% B, 15–17 min 90% B. The column was equilibrated for 6 min before each run, the total run time was 23 minutes. Flow rate and injection volume were set to 0.450 mL min^−1^ and 2.5 μL, respectively. All solutions and standards were made in 40% MeCN to make injection of solvent as the same composition of the mobile phase in the LC system.

#### 2.4.2 Mass spectrometry

MS was operated on a Waters Xevo TQD triple quadrupole mass spectrometer with electrospray ionization in positive ion mode. The parent ions and product ions (*m*/*z* 120.08 or 138.09) for retronecine, (*m*/*z* 168.10) for otonecine and (*m*/*z* 124.08) for platynecine type were used, no further qualifier adduct was included, as the product ions were reproducible to quantify. In the source voltage, 12 cone voltages (CV) were tested in the range 10–90 V. In the temperature source, 7 desolvation temperatures were used; 300–650 °C. In the source gas flow, 14 cone flows were tested; 5–100 L. In the mass analyzer, 12 collision energies (CE) was tested; 10–90 eV, to induce fragmentation and facilitation of monitoring product ions. The remainder of MS parameters were optimized manually. Extracted ion chromatograms (EIC) was obtained individually for all parameters, and the signal-to-noise (S/N) ratio calculated.

To verify the method development approach, individual standard solution of heliotrine, senecionine, jacobine and senkirkine with 1000 μg L^−1^ were injected directly into the MS, using the Intellistart (Waters, Milford) software. The approach worked and helped to protect the MS from direct injections of high concentration of standard solutions for many times.

After optimizing the MS parameters, MS/MS functions and one full scan were performed. In the MS/MS MRM mode, the ion traces was obtained for apex retention time (*t*_R_) ± 0.15 min. Corresponding CV and CE for PAs is listed in Table 1. The capillary voltage of 3.5 kV. The desolvation temperature 600 °C, desolvation gas flow 1000 L h^−1^, and cone gas flow 20 L h^−1^ was used. Data processing with MassLynx 4.0.2.3 (Waters, Milford, USA).

**Table 1:** Optimized parameters: *t*_R_, MS, MS/MS fragment ion, CV and CE for selected PAs

PA	*t*_R_ (min)	MS *m*/*z*	MS/MS *m*/*z*	CV (V)	CE (eV)
Monocrotaline	4.59	326.15	120.08, 138.09	40	40
Monocrotaline N-oxide	5.42	342.15	120.08, 138.09	35	35
Erucifoline	5.66	350.15	120.08, 138.09	40	35
Jacobine	7.18	352.17	120.08, 138.09	40	40
Jacobine N-oxide	7.89	368.16	120.08, 138.09	40	40
Retrorsine	8.15	352.17	120.08, 138.09	30	40
Heliotrine	8.78	314.19	120.08, 138.09	20	25
Heliotrine N-oxide	9.1	330.2	120.08, 138.09	25	30
Lindelofine	9.15	286.19	124.08	30	35
Lindelofine N-oxide	9.7	302.31	124.08	35	35
Senecionine	11.00	336.18	120.08, 138.09	20	30
Echimidine	11.40	398.21	120.08, 138.09	30	30
Senecionine N-oxide	11.51	352.17	120.08, 138.09	40	30
Senkirkine	11.67	366.19	168.10	35	25
Echimidine N-oxide	11.71	414.20	120.08, 138.09	30	35

### 2.5 Method validation

Method validation was conducted for five PAs (heliotine, monocrotaline, jacobine, jacobine N-oxide and senkirkine) in both soil and water matrix samples. Sandy soil from Vejle, clay loam soil from Taastrup (near Borup and Holte, Denmark), inert glass material and deionized water were used. It was tested whether interfering compounds from the soil were extracted with PAs in an experiment comparing yield of PAs from spiked soil and spiked glass fibre. For quantification, the calibration curves (9-point) were obtained using an external standard calibration. The curves were constructed by plotting the peak area *versus* the concentration of each analyte, as *y* = *ax* + *b* with weighting factor of 1/*x*, in duplicate, (Table 2), to minimize the distortion of concentration. Precision and accuracy were evaluated for intra- and inter-day variations. For intra-day variation, 3 concentration levels 25, 50 and 100 μg L^−1^ of five PAs in triplicate were spiked to matrix samples. Then, the spiked samples were extracted as described in Section 2.3 on the same day. For the inter-day variation test, new solutions were prepared in parallel and analysed for three consecutive days. Precision was calculated by relative standard deviation. The method recovery was used to calculate accuracy. Matrix effect (ME) was determined by dividing peak area of matrix samples spiked on peak area of standards. Two concentration levels (25 and 100 μg L^−1^) of five PAs were used, in triplicate. Linearity was tested by expanding calibration curve, in triplicate. To calculate the limit of detection (LOD) and limit of quantification (LOQ), 7 injection of 25 μg L^−1^ standards solution is used. They are calculated as 3 and 10 times respectively the standard deviation of peak areas divided by the slope of the calibration curve for each PAs.^27^ Overall the method was validated within limits specified in SANCO document 12495/2011.^28^

**Table 2:** Validation parameters of the final UPLC-MS/MS method

PAs	Correlation coefficient (*R*)	Sensitivity^a^ (slope)	LOD (μg L^−1^)	LOQ (μg L^−1^)	Recovery% ± SD^b^				ME%^d^	
					Soil			Water^c^	Soil	Water
					Sandy soil	Clay loam soil	Glass fiber			
Monocrotaline	0.990	*y* = 44*x*	5.5	8.7	85 ± 6	76 ± 9	94 ± 3	89 ± 10	88	94
Monocrotaline N-oxide	0.990	*y* = 40*x*	4.6	7.5	88 ± 10	72 ± 8	96 ± 4	94 ± 3	82	91
Erucifoline	0.990	*y* = 82*x*	3.0	6.3	82 ± 12	75 ± 9	92 ± 4	91 ± 8	81	95
Jacobine	0.995	*y* = 21*x*	2.6	7.6	89 ± 6	81 ± 9	96 ± 5	96 ± 6	81	91
Jacobine N-oxide	0.993	*y* = 40*x*	4.7	6.2	91 ± 7	80 ± 7	97 ± 5	93 ± 7	90	91
Retrorsine	0.991	*y* = 52*x*	6.5	9.3	80 ± 13	80 ± 7	90 ± 4	96 ± 6	91	93
Heliotrine	0.989	*y* = 6*x*	6.9	9.6	81 ± 11	78 ± 12	96 ± 4	91 ± 8	85	89
Heliotrine N-oxide	0.992	*y* = 11*x*	4.2	7.6	83 ± 9	76 ± 10	98 ± 3	90 ± 6	86	93
Lindelofine	0.988	*y* = 14*x*	6.2	9.6	86 ± 8	80 ± 9	96 ± 2	91 ± 8	89	90
Lindelofine N-oxide	0.990	*y* = 22*x*	5.6	8.5	88 ± 12	78 ± 8	97 ± 4	92 ± 5	89	91
Senecionine	0.990	*y* = 22*x*	5.4	9.7	90 ± 7	68 ± 9	97 ± 3	91 ± 7	89	98
Senecionine N-oxide	0.991	*y* = 227*x*	5.6	9.5	81 ± 12	73 ± 6	90 ± 5	95 ± 6	80	96
Echimidine	0.980	*y* = 78*x*	4.5	8.1	89 ± 8	80 ± 9	93 ± 5	90 ± 5	87	91
Senkirkine	0.994	*y* = 99*x*	5.0	5.8	82 ± 9	65 ± 9	89 ± 7	95 ± 4	82	95
Echimidine N-oxide	0.986	*y* = 96*x*	5.1	9.6	88 ± 7	78 ± 8	94 ± 6	91 ± 5	86	92

a Average of linear regression of three injections of all PAs.

b Average recovery ± standard deviation.

c De-mineralized water.

d ME = matric effect.

## 3. Results and discussion

**Fig. 2:**
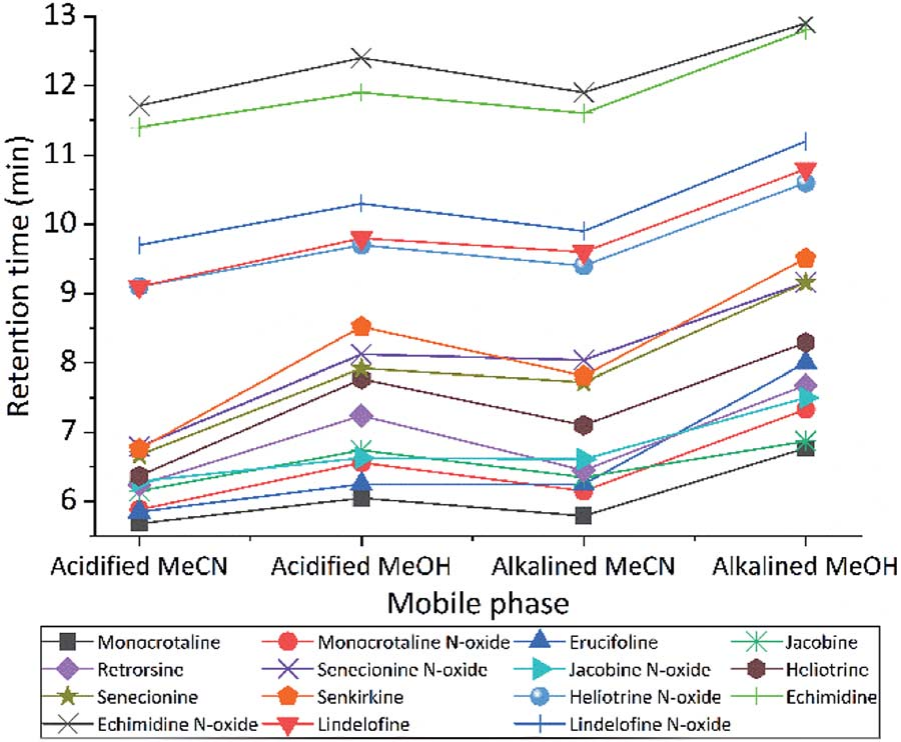
The impact of pH and additives on *t*_R_ of PAs. The sequence was running at flow rate 0.45 mL min^−1^, gradient 3. Acidified MeCN and MeOH are acidified to pH = 2.7 with FA, also alkalined MeCN and MeOH are basic to pH = 8 with NH_4_OH with NH_4_HCO_2_. Unless specified the LC parameters used as final version of optimized method.

We tested different mobile phase and pH to choose optimal one for LC separation, MeCN and MeOH with two pH (2.7 and 8) were tested (Fig. 2). During separation, the acidic MeCN yielded by far best separation. Acidic condition improve full width at half maximum and increase the peak capacity (from 22 to 93 peaks per gradient) during the separation gradient. We decided to add 1% FA for both mobile phases. Elution time of PAs did not change with pH, but the retention times were longer for MeOH eluents (both for acidic or basic) compared to MeCN. The retention factor (*k*) for both solvents was in the range 1–5, however MeOH always had higher *k* value indicating that PAs are retained more and has spent more time interacting with the stationary phase. The *k* data helped to obtain the optimum resolution, by having resolution value of 1.5 or greater between two peaks, to ensure that the sample components are well separated to a degree at which the area or height of each peak may be accurately measured. Separation with MeCN eluents produce sharper peaks, less tailing, more stable baseline, higher S/N ratio, and 2.5 fold higher peak capacity. In addition, MeCN and MeOH with different buffer systems and modifiers (FA and NH_4_OH with NH_4_HCO_3_) were tested. For ammonium formate buffers, ammonium adducts for all PAs were checked, by assuming the concentration of ammonium in the eluent correlated with ammonium adducts. However, they are not correlated. The response was higher with low concentration (1 mM) of ammonium formate, it seems the fragmentation in collision cell is suppressed by increasing the concentration of ammonium formate in the mobile phase. Then to generate protonated adduct of PAs, 0.1% FA showed higher S/N ratio and precision was two times higher than the weakest buffer system.

**Fig. 3:**
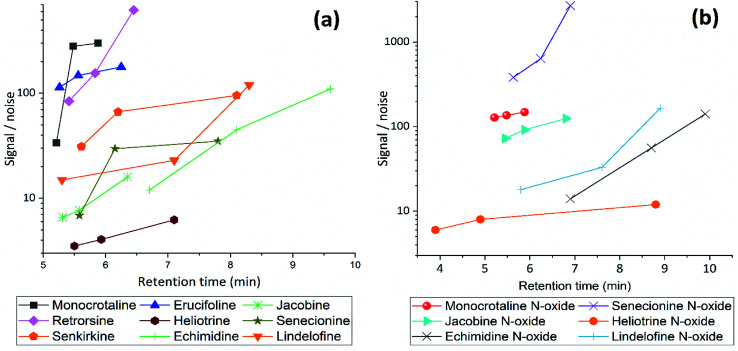
The effect of gradient condition on Signal/Noise ratio of (a) free PAs and (b) PANOs, using gradient 1, 2 and 3 with acidified MeCN (pH = 2.7), flow rate 0.45 mL min^−1^.

Subsequently, different gradients were used to choose the optimal gradient time and determine the effect of gradient on S/N ratio. Acidified (pH = 2.7) MeCN is used with gradient 1, 2 and 3. The longer gradient the higher S/N ratio for all PAs (Fig. 3). Lastly with some modification gradient 3 were chosen for chromatographic separation. Using longer gradient didn't show further improvements. For chromatographic separation, no co-eluting compounds were detected for all PA standards and samples.

Correlation coefficient (*R*), regression equation, LOD and LOQ for all PAs are listed in Table 2. Overall method detection limits are estimated to be about 2–7 μg L^−1^ for corresponded PAs, including sample preconcentration and recoveries, even this is comparable to other reported LC-MS/MS method.^1,15,29–31^ LOD can be improved by using larger amount of sorbent and dissolving samples in less than 1 mL of solvent before injection. All PAs showed a good linearity; they were linear from LOD up to 1000 μg L^−1^. MS/MS parameters optimized to increase the response and S/N ratio. CV allows to ionize the compound but does not induce fragmentation in electrospray ionization and can be used to monitor parent ion. It varied with PA type and structure (see ESI Fig. S1 and S2†), ranging from 20–40 V. With the optimal CV, the CE is changed to produce product ions in MRM run.^32^ CEs were used to investigate fragmentation patterns and intensities (see ESI Fig. S3 and S4†) in collision cell. Optimal CV and CE are listed in Table 1. Desolvation temperatures and cone flows optimized to 600 °C and 50 L, respectively. Their effect were linear on instrument's response and peak area.

Two types of sorbents has been employed to clean-up PAs, which are non-polar phases such as octylsilane or octadecylsilane [C8]^32–34^ or [C18],^35^ and cation-exchanges phases.^14–17,30^ Cation-exchange sorbent is widely used based on the suitability to retain base and PA like compounds. Therefore Oasis MCX was selected as a SPE cartridge. Demineralised water (100 mL) was spiked with 100 μg L^−1^ of PAs, and they were percolated at 10 mL min^−1^ through the cartridges. Under these conditions, the recoveries ranged between 60% and 85%.

The pH effect of water was evaluated by adjusting the samples (pH: 3, 5.5, 7, 8.5 and 10), with aliquots of FA and NH_4_OH. For all PAs, the efficiency of the extraction decreases dramatically when the pH increases except for monocrotaline, illustrated in Table 3. All PANOs exhibited high recovery at low pH. However, at high pH all PANOs showed low recovery, meanwhile corresponding free base PAs were still satisfactory, this may be due to the hydrolysis of PANOs to corresponding PAs at high pH. PAs with closed ester of retronecine, otonecine and platynecine showed better recovery at low pH.

**Table 3:** Influence of different pH of water sample on PAs recovery (%), extraction performed by loading 100 mL of water spiked with PAs 100 μg L^−1^ on MCX SPE cartridge

PAs	pH of distilled water used as loading solution				
	pH 3	pH 5.5	pH 7	pH 8.5	pH 10
Monocrotaline	89	88	75	83	93
Monocrotaline N-oxide	85	88	95	5	3
Erucifoline	91	90	78	79	86
Jacobine	87	85	64	67	57
Jacobine N-oxide	86	79	69	13	11
Retrorsine	84	89	82	74	75
Heliotrine	91	81	81	57	62
Heliotrine N-oxide	89	89	55	30	25
Lindelofine	90	84	48	28	14
Lindelofine N-oxide	93	86	61	32	21
Senecionine	85	64	75	70	83
Senecionine N-oxide	94	89	82	74	60
Echimidine	94	81	67	58	50
Senkirkine	91	81	83	74	71
Echimidine N-oxide	92	87	68	53	44

For acid wash step, hydrochloric acid, sulfuric acid and FA were used with different concentration (0.01, 0.05, 0.065, 0.1 and 0.2 M). FA (0.065 M) showed better response, and the baseline noise very much reduced. In organic wash step, MeOH, MeCN were used different concertation and volume. MeOH (50%, 5 mL) showed better response. For PA elution, different concentrations of NH_4_OH (5, 7.5, 10 and 12.5%) in MeOH (1 : 3, 1 : 2 and 1 : 1, (v/v)) and different volumes (5, 7.5, 10, 15 mL) were used. The solution of (10% NH_4_OH 1 : 3 MeOH (v/v)) with 10 mL can simultaneously elute all PAs with recoveries ranging from 84% to 95%. The SPE method improved S/N ratio and decreased baseline noise. The recovery of the methods is increased compared with reported methods, for retronecine, otonecine and heliotridine types by 14%, 30% and 59%, respectively.^10,17,24^

**Fig. 4:**
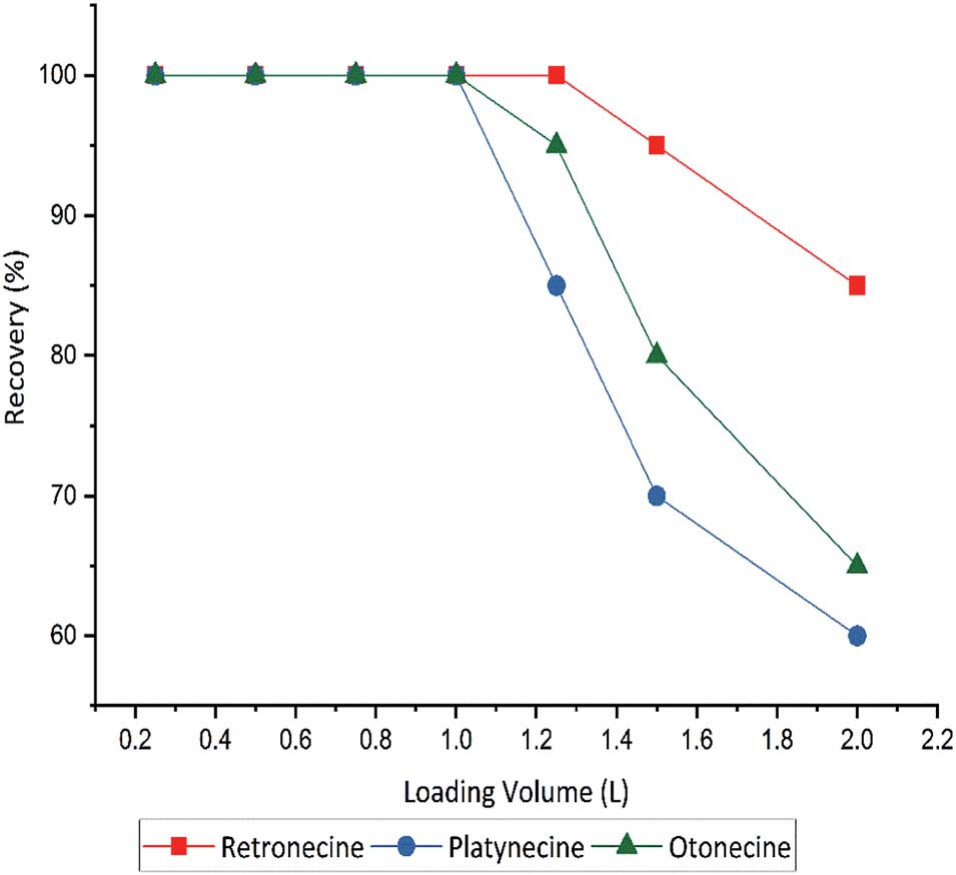
Influence of loading volume on recovery yields of three PA types retronecine, otonecine and platynecine for the optimised SPE method with MCX cartridge (150 mg), and total spiked amount 100 μg L^−1^ of each compound.

The sensitivity of the methods was improved by using different loading volume (0.25, 0.5, 1, 1.25, 1.5 and 2 L), to maximize the sample volume without including any breakthrough. Full recovery of PAs was obtained until 1.0 L sample volume, hereafter that recovery decreased. A breakthrough was observed when the loading volume exceeded 1000 mL. Recovery of three PA types in different sample volumes shown in Fig. 4.

### 3.1 Application on environmental samples

PAs were determined in environmental samples of soil and surface water, their concentrations listed in Table 4. To our knowledge, the concentration of PAs in water and soil are reported for the first time here, therefore no comparison can be drawn. The concentration of PAs in water and soil possibly correlated with the PAs in ragwort as there is abundant vegetation at the sample locations. In the soil samples, more than 86% of the 15 PAs were quantified, however in water 60% of the PAs were quantified. Thus, may explain that the PAs released from the plants into the soils, and then into water possibly with degradation. Also, 17 out of 90 PA concentrations (18%) exceeded 100 μg L^−1^. In topsoil, Holte had the highest concentration of PAs; senecionine N-oxide (1349 μg kg^−1^) and seneciphylline N-oxide (600 μg kg^−1^), followed by senecionine N-oxide (514 μg kg^−1^) in Vejle and integerrimine N-oxide (438 μg kg^−1^) in Holte. In Vejle sub-soil, the PAs concentration ranged 3–370 μg kg^−1^, jacobine N-oxide is the predominant PA. However, PA concentration in surface water were lower compare to the soils, they were ranged 6–270 μg L^−1^, jacobine was the highest PA (270 μg L^−1^) followed by seneciphylline (213 μg L^−1^) and jacoline (82 μg L^−1^). Also, some of the PAs were not detected.

**Table 4:** Content of PAs in soil (μg kg^−1^) and water samples (μg L^−1^) collected at a three ragwort locations, (*n* = 3)^a^

Location	Vejle		Borup	Holte	Vejle		Standard used for quantification
Type of sample	Soil mean ± SD (μg kg^−1^)				Water mean ± SD (μg L^−1^)		
	Topsoil	Sub-soil (below 2 cm)	Topsoil	Topsoil	Pond – surface water		
Date	4. 4. 2018	4. 4. 2018	28. 5. 2018	20. 5. 2018	4. 4. 2018	11. 5. 2018	
Jacoline	4 ± 1	3 ± 1	14 ± 1	26 ± 2	34 ± 2	82 ± 2	Jacobine
Jacoline N-oxide	47 ± 8	11 ± 2	70 ± 2	82 ± 4	5 ± 1	29 ± 1	Jacobine N-oxide
Riddelline	3 ± 1	ND	ND	5 ± 1	11 ± 1	ND	Erucifoline
Erucifoline	ND	3 ± 1	4 ± 1	3 ± 1	23 ± 1	ND	Erucifoline
Seneciphylline N-oxide	134 ± 1	48 ± 2	106 ± 5	600 ± 22	ND	6 ± 1	Senecionine N-oxide
Erucifoline-N-oxide	19 ± 1	13 ± 1	74 ± 2	369 ± 12	4 ± 1	18 ± 1	Erucifoline-N-oxide
Riddelline N-oxide	3 ± 1	4 ± 1	3 ± 1	10 ± 2	ND	ND	Erucifoline-N-oxide
Jacobine	6 ± 1	6 ± 1	226 ± 4	288 ± 9	270 ± 4	122 ± 9	Jacobine
Integerrimine N-oxide	41 ± 2	17 ± 1	138 ± 3	439 ± 21	ND	6 ± 1	Senecionine N-oxide
Senecionine N-oxide	514 ± 12	103 ± 1.3	45 ± 3	1349 ± 19	6 ± 1	6 ± 1	Senecionine N-oxide
Jacobine N-oxide	141 ± 2	370 ± 5.1	91 ± 3	232 ± 8	7 ± 1	47 ± 2	Jacobine N-oxide
Seneciphylline	6 ± 1	21 ± 1	36 ± 2	92 ± 2	6 ± 1	213 ± 10	Senecionine
Integerrimine	ND	8 ± 2	6 ± 1	42 ± 2	ND	ND	Senecionine
Senecionine	22 ± 2	17 ± 1	44 ± 3	296 ± 6	ND	ND	Senecionine
Acetylerucifoline	191 ± 1	110 ± 4	22 ± 1	82 ± 10	ND	ND	Erucifoline

a ND: not detected or under LOD.

## 4. Conclusion

The new UPLC-MS/MS method was developed to quantify PAs in soil and water from the environment with limit of detection for the UPLC-MS/MS 2–7 μg L^−1^ for ten selected PAs. In the method, parameters of both LC and MS part optimized separately. The MS part operated with full scan mode and MRM mode combined in one measurement. As a result, all three types of PAs (free base and N-oxide) could be quantified concurrently, in a considerable shorter runtime compared with previous methods. In addition, full validated SPE for clean-up and pre-concentration up to 1000 times provide 90–100% recovery for different PAs. To the best of our knowledge this is the first study on measuring PAs in soil and water, as the result 15 PAs were quantified, in a range of 3 to 1349 μg kg^−1^ in soil and 4 to 270 μg L^−1^ in surface water. The method will be an efficient platform to further study PAs in natural water and aquifers, and to follow their fate after being released or washed into the environment.

## Conflicts of interest

There are no conflicts to declare.

## Supplementary Material

RA-009-C9RA05301H-s001

## References

[cit1] Avula B., Sagi S., Wang Y. H., Zweigenbaum J., Wang M., Khan I. A. (2015). Food Chem..

[cit2] Robertson J., Stevens K. (2017). Nat. Prod. Rep..

[cit3] Moreira R., Pereira D. M., Valentão P., Andrade P. B. (2018). Toxicology and Food Safety. Int. J. Mol. Sci..

[cit4] Gertruida M. R., Christo J. B., Jacobus N. E. (2014). Phytochem. Lett..

[cit5] Fu P. P., Xia Q., Lin G., Chou M. W. (2004). Drug Metab. Rev..

[cit6] Li S. L., Lin G., Fu P. P., Chan C. L., Li M., Jiang Z. H., Zhao Z. Z. (2008). Mass Spectrom..

[cit7] Kristanc L., Kreft S. (2016). Food Chem. Toxicol..

[cit8] Boppre M. (2011). Food Addit. Contam., Part A.

[cit9] EFSA (2011). EFSA J..

[cit10] Bolechová M., Cáslavský J., Pospíchalová M., Kosubová P. (2015). Food Chem..

[cit11] van Egmond H. P. (2004). Anal. Bioanal. Chem..

[cit12] BfR, Opinion No 018/2013 of 15.06.2013, 2013, https://www.bfr.bund.de/cm/349/pyrrolizidine-alkaloids-in-herbal-teas-and-teas.pdf, accessed 25th June 2019

[cit13] JOINT FAO/WHO , 2012, http://www.fao.org/tempref/codex/Meetings/CCCF/CCCF5/cf05_INF.pdf, accessed 25th June 2019

[cit14] Gottschalk C., Ronczka S., Preiß-Weigert A., Ostertag J., Klaffke H., Schafft H., Lahrssen-Wiederholt M. (2015). Anim. Feed Sci. Technol..

[cit15] Kowalczyk E., Kwiatek K. (2018). J. Vet. Res..

[cit16] Griffin C. T., Danaher M., Elliott C. T., Kennedy D. G., Furey A. (2013). Food Chem..

[cit17] Zhu L., Ruan J., Li N., Fu P. P., Ye Y., Lin G. (2016). Food Chem..

[cit18] Rodriguez-Aller M., Gurny R., Veuthey J. L., Guillarme D. (2013). J. Chromatogr. A.

[cit19] Botitsi H. V., Garbis S. D., Economou A., Tsipi D. F. (2011). Mass Spectrom. Rev..

[cit20] Clauson-Kaas F., Hansen H. C. B., Strobel W. B. (2016). Anal. Bioanal. Chem..

[cit21] Crews C., Berthiller F., Krska R. (2010). Anal. Bioanal. Chem..

[cit22] These A., Bodi D., Ronczka S., Lahrssen-Wiederholt M., Preiss-Weigert A. (2013). Anal. Bioanal. Chem..

[cit23] Kempf M., Wittig M., Reinhard A., von der Ohe K., Blacquière T., Raezke K.-P., Michel R., Schreier P., Beuerle T. (2011). Food Addit. Contam., Part A.

[cit24] Zhou Y., Li N., Choi F. F., Qiao C. F., Song J. Z., Li S. L., Liu X., Cai Z. W., Fu P. P., Lin G., Xu H. X. (2010). Anal. Chim. Acta.

[cit25] Petersson P., Frank A., Heaton J., Euerby M. R. (2008). J. Sep. Sci..

[cit26] McNaught A. D. and Wilkinson A., IUPAC. Compendium of Chemical Terminology, (the “Gold Book”), Blackwell Scientific Publications, Oxford, 2nd edn, 2014, vol. 1, ch. 1, pp. 1074–1075

[cit27] Long G. L., Winefordner J. D. (1983). Anal. Chem..

[cit28] EURL European Commission , Contract No: 26-09, 2013, https://ec.europa.eu/food/sites/food/files/plant/docs/pesticides_mrl_guidelines_wrkdoc_2017-11813.pdf, accessed on 25th June 2019

[cit29] Tang J., Cheng M., Hattori M. (2012). Anal. Methods.

[cit30] Rozhon W., Kammermeier L., Schramm S., Towfique N., Adedeji N. A., Ajayib S. A., Poppenbergera B. (2018). Phytochem. Anal..

[cit31] Kaltner F., Rychlik M., Gareis M., Gottschalk C. (2018). J. Agric. Food Chem..

[cit32] Hosch G., Wiedenfeld H., Dingermann T., Roder E. (1996). Phytochem. Anal..

[cit33] BfR, BfR-PA-Honey-1.0/2013, 2013, https://www.bfr.bund.de/cm/349/determination-of-pyrrolizidine-alkaloids-pa-in-honey.pdf, accessed 20th June 2019

[cit34] BfR, BfR-PA-Tea-2.0/2014, 2014, https://www.bfr.bund.de/cm/349/determination-of-pyrrolizidine-alkaloids-pa-in-plant-material.pdf, accessed 20th June 2019

[cit35] Mroczek T., Glowniak K., Wlaszczyk A. (2002). J. Chromatogr. A.

