# Supplementary material for: Pyrrolizidine alkaloids quantified in soil and water using UPLC-MS/MS
Source: RSC Adv. 2019 Sep 25;9(52):30350–7. doi: 10.1039/c9ra05301h (PMC9072114; doi:10.1039/c9ra05301h)
Supplement: RA-009-C9RA05301H-s001 [file RA-009-C9RA05301H-s001.pdf]

Electronic Supplementary Material (ESI) for RSC Advances.
This journal is © The Royal Society of Chemistry 2019

Electronic supplementary information

Pyrrolizidine Alkaloids Quantified in Soil and Water using UPLC-MS/MS

Jawameer R. Hama, Bjarne W. Strobel

Department of Plant and Environmental Sciences, University of Copenhagen, Thorvaldsensvej 40, 1871 Frederiksberg, Denmark

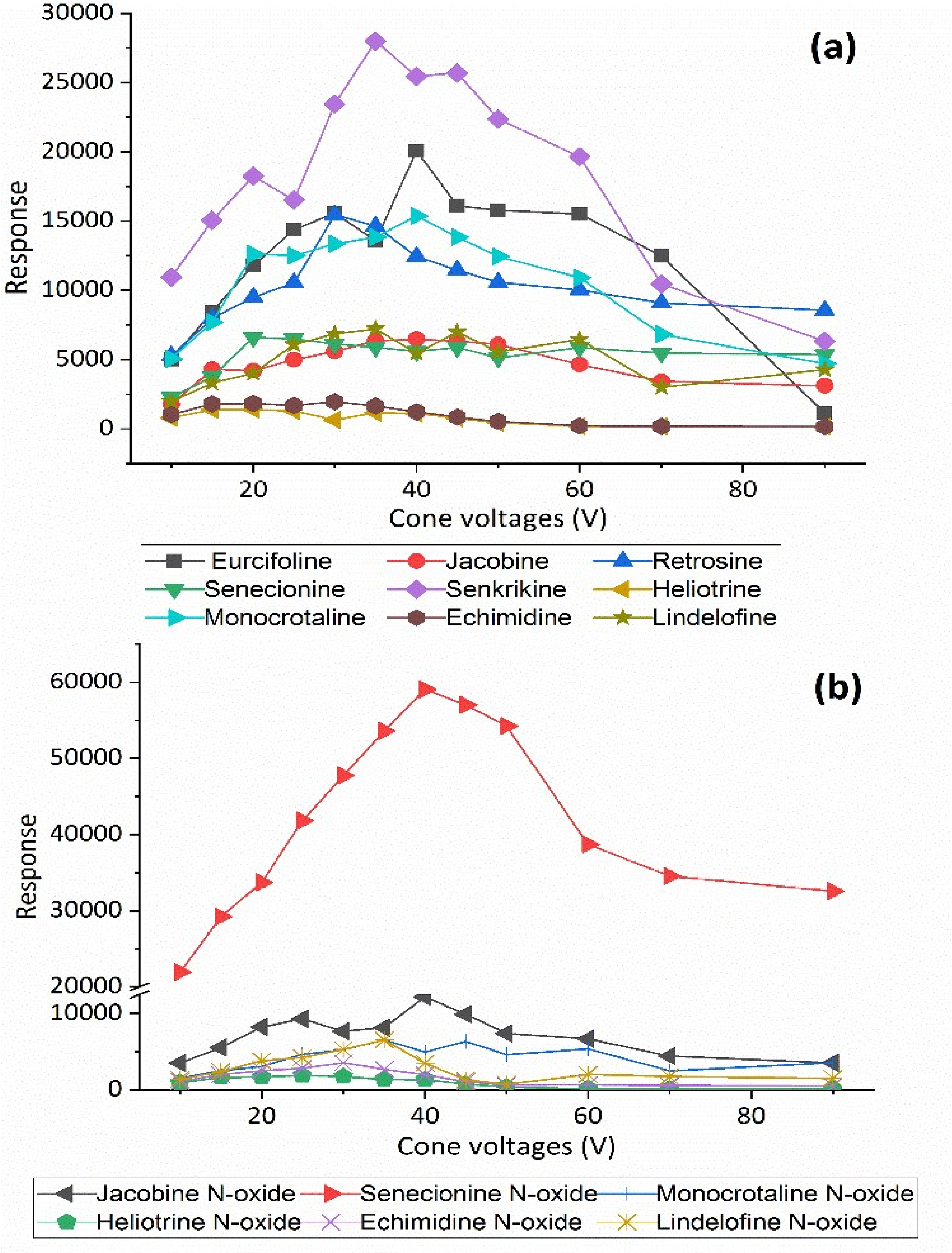

Fig. S1. The effect of different cone voltages, 10-90 V on the instrument response for (a) free PAs and (b) PANOs.

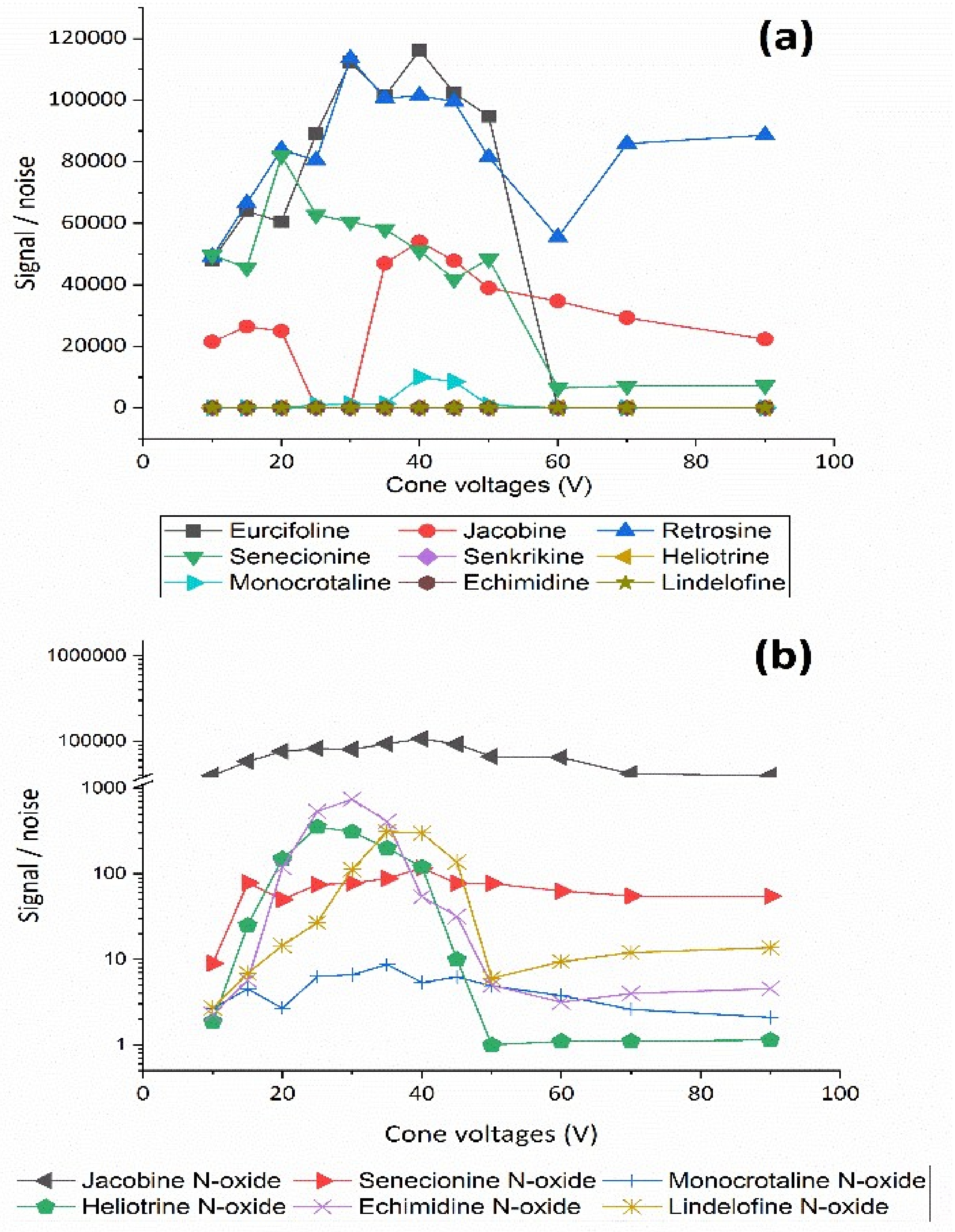

Fig. S2. The effect of different cone voltages, 10-90 V on the signal/noise ration for (a) free PAs and (b) PANOs.

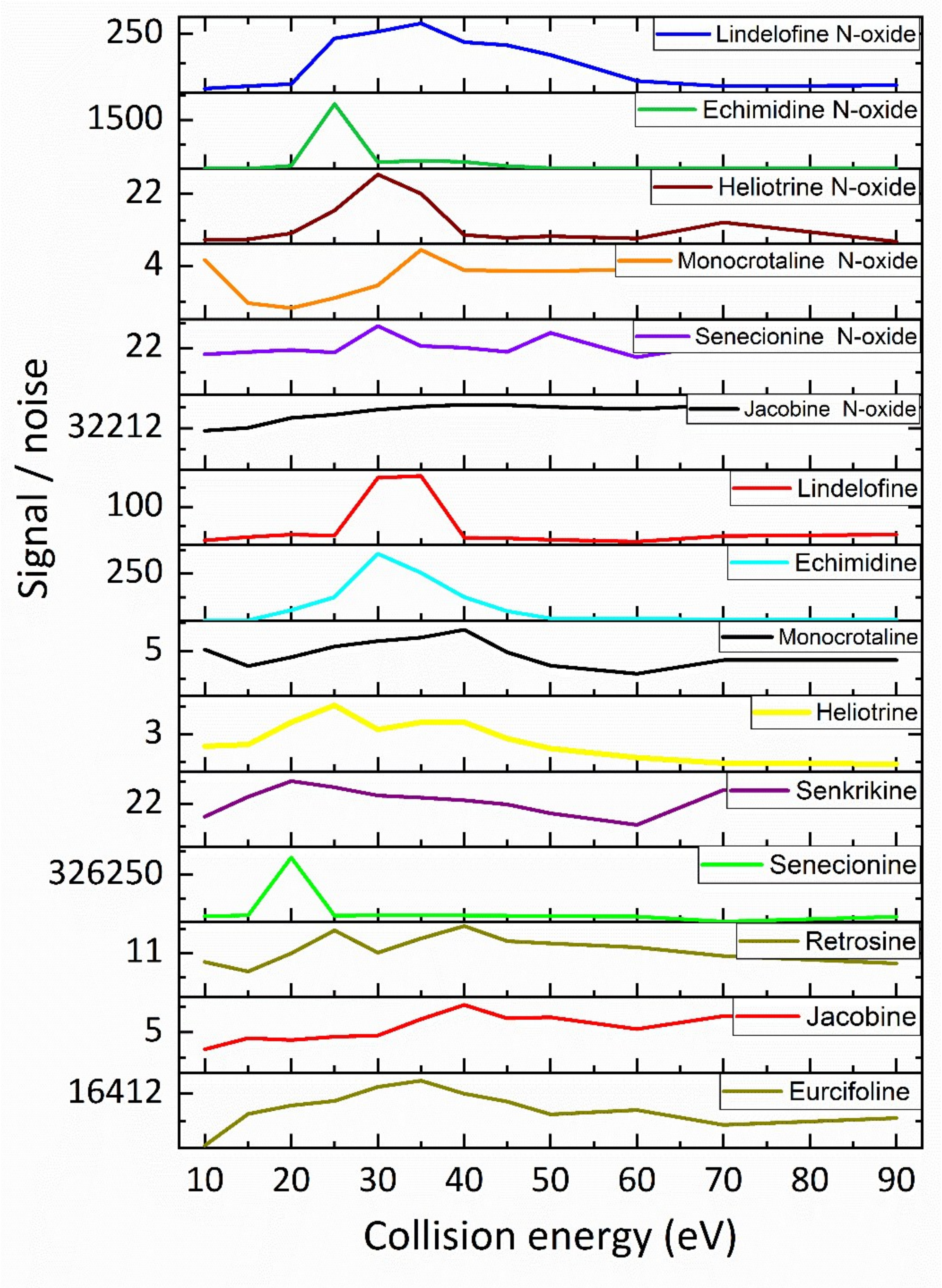

Fig. S3. The effect of different collision energies 10-90 eV on signal to noise for selected PAs.

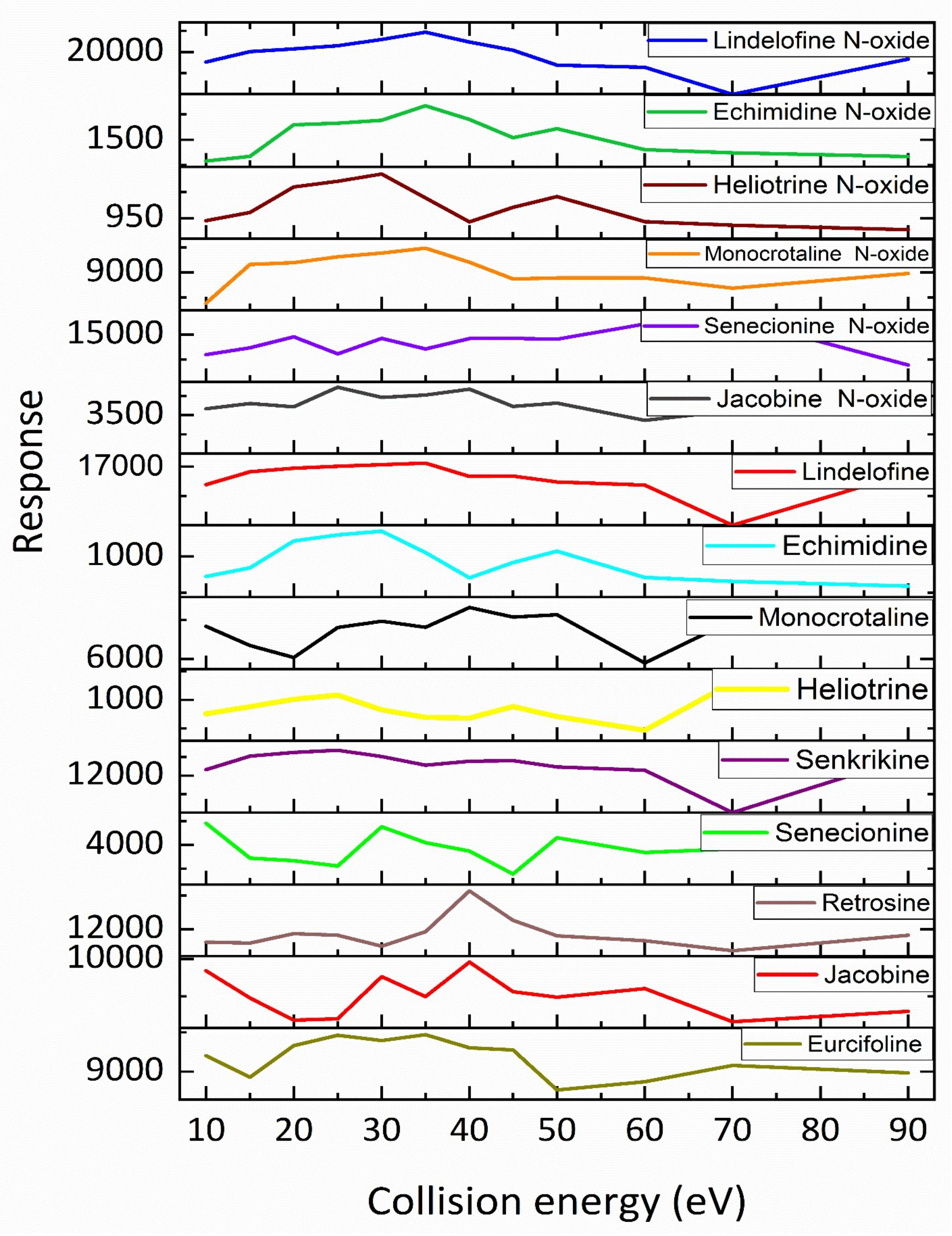

Fig. S4. The effect of different collision energies 10-90 eV on the instrument response for selected PAs.
